# Fertility Sparing Surgery in Young Woman With Granulosa Cell Tumor Followed by a Successful Pregnancy

**DOI:** 10.7759/cureus.35359

**Published:** 2023-02-23

**Authors:** Abhishek Sonkusare, Prachi Dixit, Pragati J Karmarkar, Dipanjali Thombare

**Affiliations:** 1 Department of Obstetrics and Gynaecology, NKP Salve Institute of Medical Sciences and Research Centre and Lata Mangeshkar Hospital, Nagpur, IND; 2 Department of Pathology, NKP Salve Institute of Medical Sciences and Research Centre and Lata Mangeshkar Hospital, Nagpur, IND

**Keywords:** case report, successful pregnancy, granulosa cell tumour, young woman, fertility sparing surgery

## Abstract

An uncommon form of cancer known as a granulosa cell tumor (GCT) arises from ovarian sex cord cells that produce estrogen. The goal of conservative surgery in cancer is to retain organ functions while avoiding severe excision wherever possible. In oncologic gynecological surgery, fertility-sparing surgery (FSS) is a technique that tries to preserve the uterus and ovarian tissue. A 26-year-old woman with nulligravida presented with the main complaints of pain in the right iliac fossa for 10 to 15 days and fullness in the abdomen for one month, along with a change in appetite and noticeable weight loss. The Magnetic Resonance Imaging (MRI) revealed a large, well-defined, multiloculated, solid cystic mass with altered signal intensity. On exploratory laparotomy, an intraoperatively left ovarian cystic mass was seen. The ovarian mass was histopathologically diagnosed as a sex cord tumor of the ovary, with characteristics compatible with adult GCT. Disregarding the follow-up advice on discharge four months later, the patient conceived spontaneously and gave birth to a male child via emergency lower segment cesarean section. In GCTs that have not spread beyond the ovary or in people who have had relapses of the disease, FSS created the groundwork for conception and appeared safe. In the lack of any compelling supporting evidence, the line of care of terminal surgery should always be thoroughly discussed with the patient and advised for women after their families are complete.

## Introduction

Granulosa cell tumor (GCT) is an uncommon type of cancer that develops from estrogen-secreting ovarian sex cord cells. It compensates for more than 70% of sex cord-stromal tumors (SCST) but less than 5% of ovarian tumors. Adult GCT (AGCT) and juvenile GCT (JGCT) are two separate histological forms that exhibit various histopathological and clinical characteristics [1]. With the highest incidence rate at 50-55 years, AGCTs are more prevalent and typically encountered in perimenopausal and postmenopausal women. JGCTs are uncommon tumors that comprise about 5% of all GCTs and develop in young females and premenarchal women. Most GCTs are detected in the early stages and have a fair prognosis since they frequently exhibit hyperestrogenism; however, when detected at a later stage, it is associated with a poor prognosis [2]. The conventional treatments include hysterectomy via laparotomy as per the staging of carcinoma, bilateral salpingo-oophorectomy, pelvic and para-ovarian lymphadenectomy, omentectomy, and peritoneal biopsy, but since more than 10% of patients may present as young females, there is a need for fertility-sparing surgery (FSS) in the early stages of the disease [3].

In oncology, functional and conservative surgery aims to preserve the functionality of an organ and, when possible, avoids radical resection. The goal of FSS, which is increasingly employed in oncologic gynecological surgery, is to preserve uterine and ovarian tissue [4]. The use of chemotherapy in GCT is not well supported by research. When combined with adjuvant chemotherapy, SCST with advanced or recurring stages has been reported to respond between 37% and 83% of the time [5]. This case was unique as the patient presented with a mass corresponding to 36 weeks of gravid uterus size without any sign of hyperestrogenism. As intraoperative findings were favorable, the decision to have fertility-sparing surgery was taken.

## Case presentation

A 26-year-old woman who had been married for a year and was nulligravida presented with complaints of abdominal fullness for a month and pain in the right iliac fossa for 10 to 15 days. She also had a history of significant weight loss which involved 5 kg weight in six months (>5% of weight loss in six months) but had a normal appetite with no other constitutional symptoms. On examination, she was found to be obese, with a weight of 72 kg and a Body Mass Index of 32 kg/mtr2. She was pale and had a pulse rate of 100 beats/min with raised blood pressure (160/80 mmHg). The abdomen was distended up to the xiphisternum with a mass corresponding to a 36-week gravid uterus size, and it was regular and firm in consistency. However, there were no signs of lymphadenopathy or edema.

The patient did not give consent for speculum examination. On per vaginal examination, fullness was present in the anterior fornix, which was cystic in consistency. The cervix deviates to the right side. Uterus size couldn’t be made due to the size and tenderness of the mass, and uterine mobility was also restricted. The ultrasonography (USG) revealed a large cystic lesion of size 23 x 27 x 26 cm with a volume of 8763 ccs with multiple internal septations within it, occupying the abdominopelvic cavity. The RMI score was 38 (25-250 >20% risk of malignancy). The provisional diagnosis on USG was of neoplastic etiology.

The patient was admitted for further evaluation of the abdominal mass. The Magnetic Resonance Imaging (MRI) revealed a large, well-defined, multiloculated, solid cystic mass with altered signal intensity, measuring 15 x 23 x 25 cm in size, suggestive of a well-defined, large, multiloculated, multiseptated lesion with the following differential diagnoses: left ovarian dysgerminoma, large abdominal echinococcal cyst, or a mesenteric lymphangioma.

Her biochemical evaluation showed a reduced hemoglobin of 7.1gm/dl, and her hematocrit value was 20.90%. Her CA-125 was 38.15 units/ml and her serum lactate dehydrogenase slightly raised to 354U/L, her beta Human Chorionic Gonadotropin level was <0.10mIU/ml, and her serum inhibin A value was 7.2 pg/ml as shown in Table 1. The patient tested negative for Echinococcus (hydatid cyst) IgG. Preoperatively over five days, the patient received a blood transfusion with three bags of packed red cells (PRC) as a treatment for anemia. The preoperative hemoglobin of the patient was 9.6gm/dl, as shown in Table 1. The decision of staging laparotomy with an attempt at fertility conservation was taken after discussing the need for the completion of a re-laparotomy based on the histopathology report.

**Table 1:** Laboratory results with reference range.

Parameters	Laboratory Values	Normal Range
Haemoglobin	7.1 gm/dl	11.6 to 15 gm/dl
Pre-operative haemoglobin	9.6 gm/dl	11.6 to 15 gm/dl
Hematocrit	20.90%	37% to 47%
Serum lactate dehydrogenase	354 U/L	122 TO 222 U/L
Cancer Antigen-125 (CA-125)	38.15 units/ml	0-35 units/ml
Beta human chorionic gonadotropin (HCG)	<0.10 mlU/ml	<5 mlU/ml is considered negative for pregnancy
Serum Inhibin A	7.2 pg/ml	2-80 pg/ml

**Figure 1:**
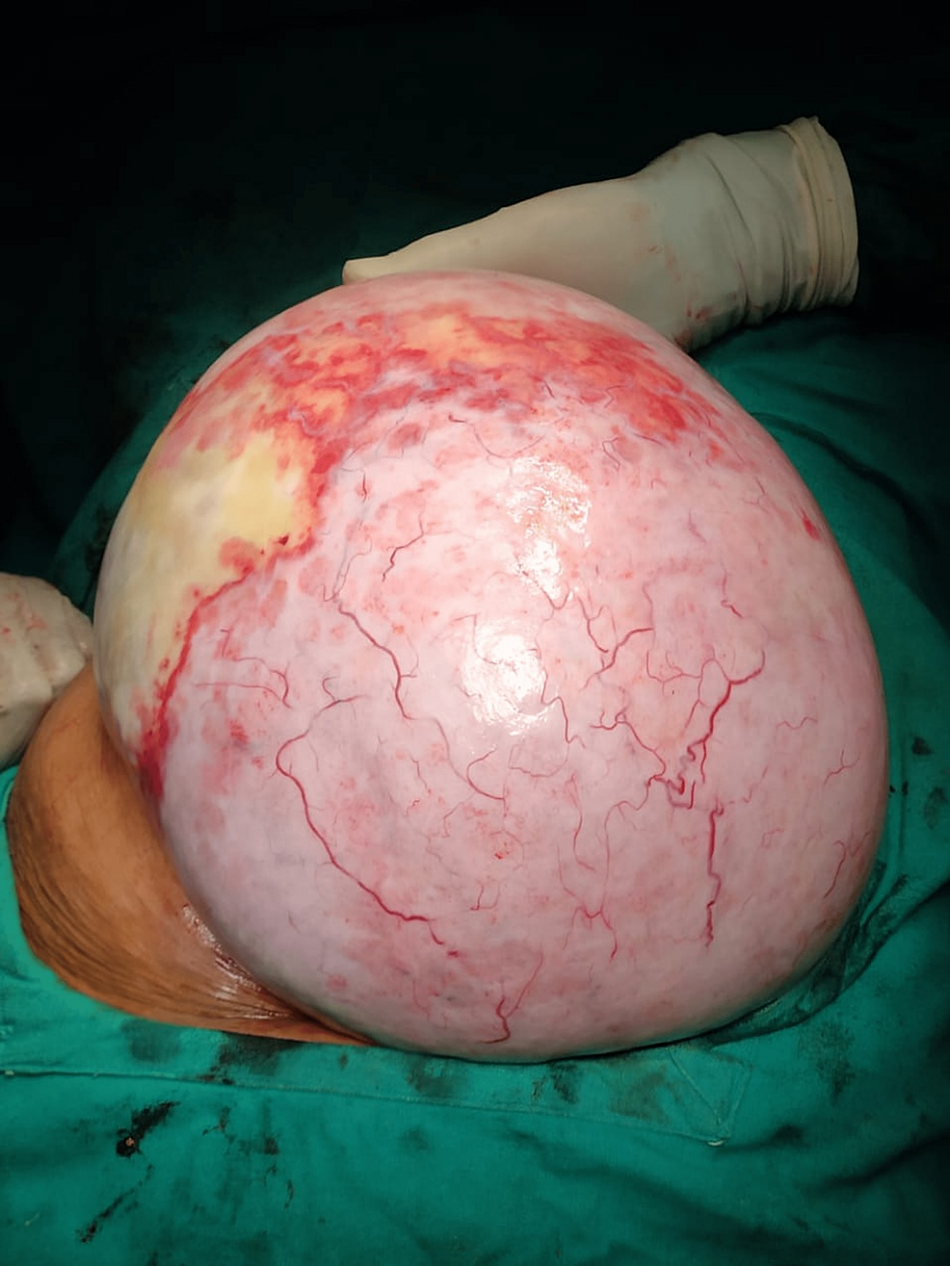
Ovarian cystic mass with smooth surface and intact capsule

**Figure 2:**
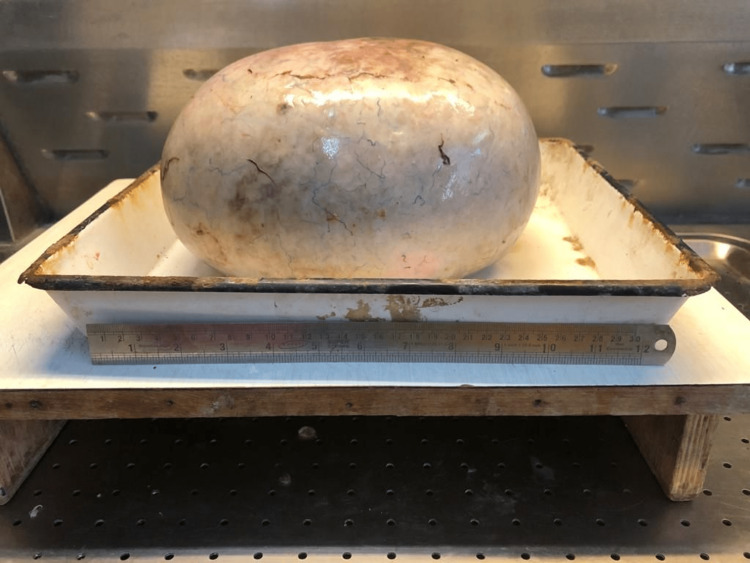
Ovarian cyst mass

**Figure 3:**
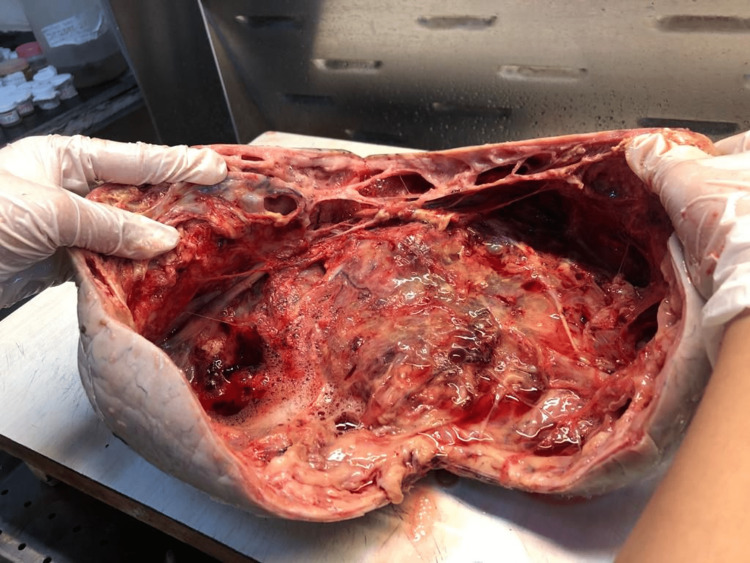
The cross-sectional cut of ovarian cyst

**Figure 4:**
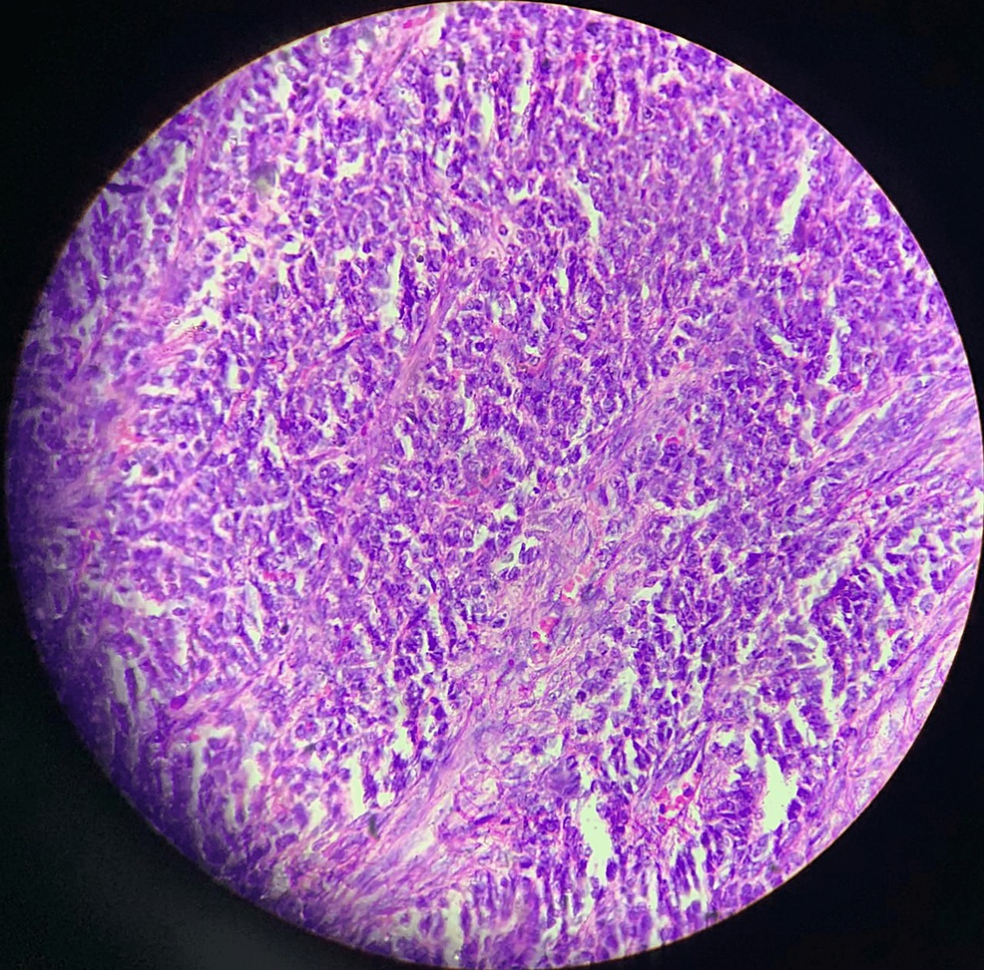
Histopathology of the ovarian mass showing sex cord tumor of the ovary

On exploratory laparotomy, an intraoperatively left ovarian cystic mass of 30 x 26 x 27 cm was seen with a smooth surface and intact capsule, as shown in Figures 1, 2. No ascites were present, and the second right ovary was normal. The postoperative course was uneventful, and the patient was given a blood transfusion with one bag of PRC. The specimen was cut in the pathology lab, after which the histopathology examination of the ovarian mass showed a sex cord tumor of the ovary, with findings consistent with AGCT, as shown in Figures 3, 4. The staging was 1A1 according to the FIGO classification. There is no need for a biopsy of the contralateral ovary as the disease is unilateral in 98% of the cases.

The patient had been advised to follow up with MRI and inhibin levels for up to two years and advised for using strict contraception. Injection of depot-medroxyprogesterone acetate (DMPA) was planned at follow-up visits, but she disregarded the advice on discharge, and four months later, the patient conceived spontaneously and arrived for antenatal care (ANC) and followed regular ANC visits to the hospital. She was admitted as a primigravida with a Comprehensive Gestational Age (CGA) of 37 weeks-2 days with chronic hypertension. The patient underwent an emergency lower segment cesarean section given severe preeclampsia with a poor Bishop score, and the patient was not willing to trial labor and delivered a male infant by cesarean. There was no evidence of recurrence of granulosa cell tumor at the time of delivery. She has not shown any indicators of recurrence for the past 14 months since her initial diagnosis.

## Discussion

Different signaling routes are needed for granulosa cell proliferation, and any alteration to these signaling pathways results in excessive granulosa cell proliferation and the development of GCT. Understanding the role of these pathways in the etiology of the disease allows for the adoption of novel treatments to treat GCT, especially in the case of recurrent GCT [6]. The FOXL2 gene expresses the transcription regulator necessary for the granulosa cell to mature normally. A somatic translational mutation in FOXL2 was found in GCT by Shah et al. Due to its high frequency, this mutation may be pathognomic for AGCT, and its lack of JGCT may indicate the presence of a completely distinct tumor [7,8].

Wang et al., in a study, concluded for young patients who want to protect their fertility, FSS is a viable alternative. In patients with an unstaged AGCT, secondary surgical staging is an important form of treatment [9]. The success of proliferation is remarkable. If they are willing to undertake a protracted follow-up, radical surgery may be postponed till recurrence [10]. Chan et al. undertook a study that examined the prognostic parameters responsible for survival in ovarian sex cord-stromal tumors (SCST) and found that less than 50 years CGA, lower tumor size, and the absence of residual disease are key indicators of improved survival in patients with SCST of the ovary [11].

Lee et al. conducted a study on clinicopathological characteristics of GCT of the ovary and concluded that the sole component related to disease survival is the stage of the disease; therefore, FSS may be a therapy option for women with early-stage disease who want to keep their fertility [12]. Similar to this, a retrospective study given by Ayhan et al. studied the prognostic factors in adult GCT and concluded that a thorough staging procedure should be undertaken to determine the enormity of the disease and to more accurately predict the oncologic prognosis. The initial stage appears to be the single most important prognostic factor in ovarian GCT [13].

Rema et al., in their study, stated that after FSS, chemo-induced gonadotoxicity compromises fertility when a patient requires chemotherapy in adjuvant. By employing GnRH analogs or oral contraceptives to downregulate hormones, the surviving ovarian tissue can be somewhat safeguarded. In their first or second decade of life, girls are most susceptible to germ-cell ovarian cancers. They typically have the early disease when they first present, making them candidates for fertility preservation. Comprehensive surgical staging also includes a unilateral salpingo-oophorectomy. Bleomycin, etoposide, and cisplatin (BEP) are used in chemotherapy to treat extra ovarian illness. Despite the fact that chemotherapy increases the chance of ovarian failure, most patients after BEP resume menstruation and have good reproductive outcomes [14].

## Conclusions

FSS appears to be a safe treatment option for people with GCTs and offers a possibility for women with early-stage disease who want to maintain their fertility. The degree of care for termination of surgery should always be thoroughly discussed with the patient and recommended for all women after their families are complete in the absence of any convincing supporting data.
